# Factors Associated with Antiretroviral Therapy Adherence Among Adolescents Living with HIV in Eastern Cape Province

**DOI:** 10.3390/ijerph22111718

**Published:** 2025-11-13

**Authors:** Onesimo Maxolo, Laston Gonah, Ziphelele Ncane, Guillermo Alfredo Pulido-Estrada

**Affiliations:** Department of Public Health, Faculty of Medicine and Health Sciences, Walter Sisulu University, Mthatha 5100, South Africa; 214034887@mywsu.ac.za (O.M.); zncane@wsu.ac.za (Z.N.)

**Keywords:** antiretroviral therapy adherence, antiretroviral therapy literacy, adolescents living with HIV, viral suppression, status disclosure, treatment buddies

## Abstract

Suboptimal ART adherence contributes to treatment failure and is common among adolescents. The study sought to assess factors associated with antiretroviral therapy (ART) adherence among adolescents living with HIV. This was a retrospective cross-sectional study design with a random sample of 137 records of adolescents aged 10–19 years from four high-volume ART sites (35 records per site). Data was collected using a customized data extraction tool. Descriptive statistics and Fisher’s exact tests were used to analyze data and identify factors associated with ART adherence at 5% significance level. Most participants (80.3%) were females. Females were more likely to access ART literacy information than males (*p* = 0.033); poor ART outcomes were prevalent, characterized by high unsuppressed viral loads (73%), low status disclosure (49.6%), inadequate treatment support-lack of treatment buddies (51.2%), missed scheduled ART-related appointments (97.1%), and low rates of follow-up visits (1.5%). Some adolescents (36.5%) rely on alternative medication for HIV treatment. In conclusion, targeted interventions must focus on improving ART adherence, strengthening support systems, and promoting disclosure among adolescents living with HIV, including addressing the use of unproven HIV therapies.

## 1. Introduction

Adolescence is a period of mental, physical, and emotional maturation where an individual may undergo behavioural experimentation, identity formation, risk-taking, and face difficult choices on romantic relationships, sexual behaviour, and alcohol and recreational drug use [1]. Adolescence is sometimes characterized by high-risk sexual behaviours and a lack of engagement with healthcare services that can affect adherence to antiretroviral therapy [1].

Antiretroviral therapy adherence is a principal determinant of virologic suppression. Suboptimal adherence can include treatment interruption and discontinuation of ART, missed or late dosage intake, and subtherapeutic or partial dosing. In 2019 there was an estimated 1.7 million adolescents aged 10–19 living with HIV (ALHIV) globally, with the majority (80%) living in Sub-Saharan Africa (SSA) [2]. South Africa had the highest number of ALHIV in 2019, with an estimated 360,000 ALHIV [2].

HIV-related deaths among adolescents surged between 2019 and 2020 [1]. Despite the progress in ART, adolescents continue to experience disproportionately high rates of poor antiretroviral treatment (ART) outcomes, hindering global efforts to achieve the UNAIDS 95-95-95 targets [2]. Notably, HIV remains a leading cause of death among adolescents, with retention in care rates declining over time, from 88% at 12 months to 67% at 36 months, particularly in low- and middle-income countries [2,3]. The persistence of HIV among adolescents is a pressing public health concern, with over 250,000 new infections globally in 2019, disproportionately affecting those living in sub-Saharan Africa [4]. Non-adherence among adolescents remains a critical issue, despite the significant reduction in mortality and morbidity rates due to ART [5].

Despite progress in ART rollout and the resultant reduction in HIV incidence in South Africa, adolescents, particularly those in the Eastern Cape province, continue to experience relatively lower viral load suppression and adherence rates compared to adults and children [6]. Adolescent ART adherence is shaped by various interacting individual, treatment, social, and health-system factors. On the other hand, status disclosure, supportive treatment buddies/caregivers, counselling and health education, and reliable follow-up systems are associated with better viral suppression and retention in care [3,4,5,6,7]. In this study, we therefore focus on adherence-related processes, including ART literacy, disclosure, treatment support, and appointment keeping, as proximal determinants of adherence outcomes among adolescents in the Eastern Cape. This study sought to identify determinants of ART adherence among adolescents living with HIV in selected healthcare facilities within the Eastern Cape province. The contextual research insights can inform future studies and potentially guide targeted interventions to promote ART adherence and treatment outcomes [7].

## 2. Materials and Methods

### 2.1. Study Design

The study employed a retrospective quantitative cross-sectional design. For each adolescent, variables such as counselling attendance, disclosure status, treatment support, and clinic appointment keeping were recorded based on the most recent documentation available in the patient’s file. The study, therefore, provides a cross-sectional snapshot of adherence-related factors at the last clinical record entry, rather than a longitudinal account of changes over time.

#### 2.1.1. Study Population and Sampling

The study was conducted using clinical records of HIV-infected adolescents who were registered for ART at four high-volume ART sites in the Eastern Cape Province between January 2019 and December 2024. Eligibility was restricted to records of adolescents who had been on ART for more than six months, including those on both first-line and second-line regimens. Data extraction was carried out between November 2024 and March 2025. A random sample of 140 clinical records was required to achieve a sufficient sample size for the study, considering 10% for missing data, 6.2% of ALHIV on ART [8], a 95% confidence level, and a standard error of 4%. This figure reflects ART coverage rather than overall adolescent HIV prevalence, which is likely higher but less precisely documented in the Eastern Cape Province. In each of the four facilities, 20 clinic records were selected to reach the minimum sample size required for the study. The sample size (n) of the study population was calculated considering the following aspects: Prevalence of the adolescents infected with HIV receiving ART was estimated at 6.2%.

#### 2.1.2. Inclusion and Exclusion Criteria

Only clinical records of HIV adolescents receiving ART at the four study sites in the OR Tambo district were considered. Clinical records for adolescents with comorbidities or those with less than 6 months on ART were not included in the study. The clinical records did not include details on the mode of HIV acquisition (e.g., perinatal vs. recent infection) or the exact duration of infection; thus, these factors could not be assessed.

### 2.2. Data Collection Instruments

Data was collected using a customized data extraction tool developed from standard ART monitoring indicators [5,6,7,8,9,10,11,12,13]. The tool was reviewed by two public health researchers, two HIV clinicians, and a biostatistician to establish face and content validity. Because the tool was used to abstract information from clinical records rather than to measure latent constructs, internal consistency reliability testing was not applicable.

The target variables included: (1) socio-demographic characteristics (age, level of education, marital status, gender); (2) barriers to ART adherence (income source, disclosure status, living conditions, lifestyle, schedule conflicts, treatment side effects); (3) patient education and perceptions (importance and benefits of adherence); (4) counselling and support (pre- and post-ART counselling, prior exposure to health education); (5) adherence strategies (use of reminders, treatment burden); (6) health behaviours and screening (condom use, substance use, mental health screening, TB screening); (7) traditional medicine and practices (use and discontinuation), according to age appropriateness.

### 2.3. Data Management and Statistical Analysis

Data entry and analysis were conducted using SPSS version 29 (IBM, Armonk, NY, USA). Descriptive statistics were used to summarize the data in terms of proportions, means, and standard deviations, and results were presented in tables and graphs. Chi-square or Fisher’s exact tests were applied to identify factors associated with ART non-adherence among adolescents living with HIV (ALHIV), with *p*-values less than 0.05 considered statistically significant at the 5% level.

### 2.4. Ethics and Legal Considerations

The study adhered to the ethical principles of respect for participants’ information, beneficence, non-maleficence, and justice outlined in the Helsinki Declaration throughout the study. Ethical approval was obtained from the Walter Sisulu University (WSU) Faculty of Medicine and Health Sciences Research Ethics Committee. To ensure data security and confidentiality, the data was stored in a password-protected computer and only used for research purposes.

## 3. Results

### 3.1. Participant Characteristics

A total of 137 adolescent case records were analyzed in the study, predominantly comprising females (80.3%). Most of the participants (83.9%) were aged between 15 and 19 years, with the remainder aged 10 and 14 years (16.1%).

### 3.2. Psychosocial Readiness and ART Adherence

Table 1 presents a summary of key factors that determine psycho-social readiness of adolescents in relation to ART adherence. The majority (70.1%) had attended all the required counselling sessions, with a higher proportion of females (73.6%) participating compared to males (55.6%), although the difference was not statistically significant (*p* = 0.066).

The findings show that less than half (49.6%) of the adolescents had disclosed their HIV status to someone, where a slightly higher proportion of females (51.8%) had disclosed compared to males (40.7%; *p* = 0.302). With regards to social support in the form of treatment buddies, almost half (48.9%) of the adolescents reported having a treatment buddy, with a slightly higher proportion among females (50.9%) than males (40.7%; *p* = 0.344). Clinic attendance rate among the adolescents was low, where only 26.3% reported attending the clinic regularly, with a slightly higher attendance among females (27.3%) compared to males (22.2%), although without a statistically significant difference (*p* = 0.593). The proportion of adolescents who missed scheduled clinic appointments was high (73.7%). Upon further analysis, there was no statistically significant difference regarding all the aspects of psycho-social readiness to ART adherence between younger (10–14 years) and older (15–19 years) adolescents (*p* > 0.5, respectively).

### 3.3. Antiretroviral Therapy (ART) Literacy and Related Practices

Health literacy was assessed based on prior exposure to professional health advice. All participants reported having been exposed to formal health literacy information on lifestyle behaviours, including diet, physical activity, sexual health, and alcohol, drugs, and substance use prevention.

With regards to ART literacy, there were notable gender differences in ART literacy levels, with females generally showing higher literacy levels compared to males (Table 2). Prior exposure to professional advice on the importance of ART adherence was reported by 75.2% of adolescents, where more females had been exposed to the information compared to males (*p* = 0.033). A significant proportion of study participants had not been exposed to professional health education on the reasons for taking ARVs (25.5%), the importance of ART adherence (24.8%), and its benefits (43.1%). A significant proportion (36.5%) reported using alternative medication for the treatment of HIV or related ailments, with no significant difference between both genders (*p* = 0.408) and age groups (40.0% among 15–19-year-olds, and 18.2% among 10–14-year-olds; *p* = 0.051). The nature of these additional treatments was, however, not specified during data collection, although these could include any unconventional therapies.

### 3.4. Adherence Monitoring and Support

Existing measures for monitoring and promoting adolescent ART adherence were assessed, where some gaps were revealed: 73% had unsuppressed viral loads, 94.2% lacked adherence forms, 97.1% had missed scheduled ART appointments, and follow-up visits were low (1.5%). Most of the patient files (85.4%) also lacked treatment buddy details (Table 3). Only treatment buddy support showed a significant gender difference, where females had more treatment buddies than males (*p* = 0.017), and no significant differences were observed regarding younger (10–14 years) and older adolescents (15–19 years).

## 4. Discussion

Key study findings were that (1) females were more likely than males to access ART literacy information; (2) a high prevalence of unsuppressed viral loads was observed, alongside several adherence-related challenges such as low status disclosure, inadequate treatment support (lack of treatment buddies), missed ART-related appointments, and low rates of follow-up visits; and (3) some adolescents reported relying on alternative medications for HIV treatment.

The findings highlight critical issues of significance to adolescent ART adherence and treatment success. The disparity in access to ART literacy information in favour of females may be due to several factors, including inherent differences in health-seeking behaviours between males and females, or possible differential availability of targeted interventions that focus more on girls due to their disproportionate vulnerability [9,10]. Targeted research is required to understand care preferences and determinants of ART adherence among male adolescents, informing tailored strategies to engage males in HIV care and education and improve treatment uptake and outcomes.

The observed poor ART treatment outcomes, marked by high unsuppressed, low HIV status disclosure, inadequate treatment support, missed scheduled ART-related appointments, and low follow-up rates, are quite concerning. These findings are interconnected, with greater potential for exacerbating each other. For instance, a lack of treatment support in the form of not having treatment buddies and follow-up visits can contribute to poor adherence and, consequently, poor viral suppression [11,12]. It has been shown that addressing these gaps requires a multifaceted approach that can include strengthening support systems, improving tracking of scheduled ART appointments, and counselling to promote status disclosure [4,13].

The reported use of alternative medications for HIV treatment by some adolescents is concerning, as this can undermine ART adherence, treatment efficacy, and overall health [11,14]. Further research prioritizing behavioural data generation is required to gain more insights on types, reasons, and perceived benefits of alternative HIV treatments, to inform targeted interventions for adolescents.

The study had some limitations. Firstly, it relied on self-reported data for some outcome variables, which may be subject to recall and social desirability bias. Future research should incorporate more objective adherence measures, such as pharmacy refill records, electronic monitoring devices, and viral load data, to enhance accuracy. Secondly, a cross-sectional design limits the ability to assess long-term adherence trends and the effectiveness of interventions over time. Longitudinal studies are recommended to evaluate the sustainability of such interventions. Additionally, future research should investigate the specific needs and challenges faced by various subgroups, including adolescents in rural settings and those with co-existing conditions such as mental health disorders. Finally, the lack of information on the mode of HIV acquisition, the length of infection, and duration on ART represented other limitations. These factors are likely to influence adherence patterns and treatment outcomes among adolescents and should be explored in future studies with more detailed clinical or primary data.

## 5. Conclusions

Targeted interventions must focus on improving ART adherence, strengthening support systems, and promoting disclosure among adolescents living with HIV. Healthcare providers working in HIV treatment programs should be adequately trained to equip them with the necessary knowledge and skills required to address the unique needs of adolescents living with HIV, including discussions around alternative therapies. The study underscores the need for comprehensive and tailored approaches to support adolescent ART adherence and improve HIV treatment outcomes among adolescents.

## Figures and Tables

**Table 1:** Psychosocial readiness indicators of adolescents receiving ART, by sex and age group.

Psycho-Social Readiness		Male		Female		Total		*p* Value
		#	%	#	%	#	%	
Has the patient attended all required counselling sessions?	Yes	15	55.6	81	73.6	96	70.1	0.066
	No	12	44.4	29	26.4	41	29.9	
	Total	27	100.0	110	100.0	137	100.0	
For patients younger than 14 years, did the patient attend partial disclosure sessions?	Yes	15	55.6	82	74.5	97	70.8	0.052
	No	12	44.4	28	25.5	40	29.2	
	Total	27	100.0	110	100.0	137	100.0	
For patients 15 years and older, did the patient attend full disclosure sessions?	Yes	21	77.8	90	81.8	111	81.0	0.631
	No	6	22.2	20	18.2	26	19.0	
	Total	27	100.0	110	100.0	137	100.0	
Has the patient disclosed to anyone?	Yes	11	40.7	57	51.8	68	49.6	0.302
	No	16	59.3	53	48.2	69	50.4	
	Total	27	100.0	110	100.0	137	100.0	
Does the patient have a treatment buddy?	Yes	11	40.7	56	50.9	67	48.9	0.344
	No	16	59.3	54	49.1	70	51.1	
	Total	27	100.0	110	100.0	137	137.0	
Does the patient attend the clinic regularly?	Yes	6	22.2	30	27.3	36	26.3	0.593
	No	21	77.8	80	72.7	101	73.7	
	Total	27	100.0	110	110.0	137	100.0	

**Table 2:** Gender differences in ART literacy.

Education		Male		Female		Total		*p* Value
		#	%	#	%	#	%	
Has the patient been educated about ARVs and that they are lifelong drugs?	Yes	16	59.3	86	78.2	102	74.5	0.043
	No	11	40.7	24	21.8	35	25.5	
	Total	27	100.0	110	100.0	137	100.0	
Has the patient been educated about the importance of taking ARVs at the same time every day?	Yes	16	59.3	87	79.1	103	75.2	0.033
	No	11	40.7	23	20.9	34	24.8	
	Total	27	100.0	110	100.0	137	100.0	
Has the patient been educated not to take ARVs with immune boosters?	Yes	16	59.3	87	79.1	103	75.2	0.033
	No	11	40.7	23	20.9	34	24.8	
	Total	27	100.0	110	100.0	137	100.0	
Has the patient been educated not to use traditional medication while on ARVs?	Yes	16	59.3	87	79.1	103	75.2	0.033
	No	11	40.7	23	20.9	34	24.8	
	Total	27	100.0	110	100.0	137	100.0	
Has the patient been educated that enemas and induced vomiting are contraindicated with ARVs?	Yes	16	59.3	88	80.0	104	75.9	0.024
	No	11	40.7	22	20.0	33	24.1	
	Total	27	100.0	110	100.0	137	100.0	
Has the patient been informed about side effects of ARVs?	Yes	18	66.7	88	80.0	106	77.4	0.138
	No	9	33.3	22	20.0	31	22.6	
	Total	27	100.0	110	100.0	137	100.0	
Does the patient understand the benefit of ARVs	Yes	11	40.7	67	60.9	78	56.9	0.058
	No	16	59.3	43	39.1	59	43.1	
	Total	27	100.0	110	100.0	137	100.0	

**Table 3:** Gender differences in adherence planning and ART monitoring among adolescents living with HIV.

Adherence Planning		Male		Female		Total		*p* Value
		#	%	#	%	#	%	
Does the patient file have an attached adherence form?	Yes	0	0.0	8	7.3	8	5.8	0.355
	No	27	100.0	102	92.7	129	94.2	
	Total	27	100.0	110	100.0	137	100.0	
Do the name and details of treatment buddy appear on the patient’s file?	Yes	0	0.0	20	18.2	20	14.6	0.017
	No	27	100.0	90	81.8	117	85.4	
	Total	27	100.0	110	100.0	137	100.0	
Does the information about the patient’s understanding of taking ARVs appear in the file?	Yes	14	51.9	75	68.2	89	65.0	0.111
	No	13	48.1	35	31.8	48	35.0	
	Total	27	100.0	110	100.0	137	100.0	
Are the necessary blood results recorded in patient’s file, such as VL and CD4 count?	Yes	22	81.5	95	86.4	117	85.4	0.546
	No	5	18.5	15	13.6	20	14.6	
	Total	27	100.0	110	100.0	137	100.0	
Does the patient come according to their next appointment dates?	Yes	2	7.4	2	1.8	4	2.9	0.174
	No	25	92.6	108	98.2	133	97.1	
	Total	27	100.0	110	100.0	137	100.0	
Is a patient who misses their appointment date referred for ongoing adherence counselling?	Yes	1	3.7	13	11.8	14	10.2	0.302
	No	26	96.3	97	88.2	123	89.8	
Does routine health education provision appear on the patient file?	Yes	27	100.0	110	100.0	137	100.0	-
	No	0	0.0	0	0.0	0	0.0	
	Total	27	100.0	110	100.0	137	100.0	
Does routine mental health screening appear in the patient file, and are necessary referrals performed appropriate?	Yes	26	96.3	109	99.1	135	98.5	0.356
	No	1	3.7	1	0.9	2	1.5	
	Total	27	100.0	110	100.0	137	100.0	
Is the patient up to date on follow-up visits?	Yes	1	3.7	1	0.9	2	1.5	0.356
	No	26	96.3	109	99.1	135	98.5	
	Total	27	100.0	110	100.0	137	100.0	
Are all monitoring blood results of the patients consistently within desired ranges?	Yes	6	22.2	31	28.2	37	27.0	0.532
	No	21	77.8	79	71.8	100	73.0	
	Total	27	100.0	110	100.0	137	100.0	

## Data Availability

The primary data used for the study can be accessed from the Eastern Cape Department of Health secretariat upon request at thomas.dlamini@echealth.gov.za.

## Abbreviations

The following abbreviations are used in this manuscript:

HIV	Human immunodeficiency virus
ART	Antiretroviral therapy
ARVs	Antiretroviral drugs
ALHIV	Adolescents living with HIV

## References

[1] Kim S.-H., Gerver S.M., Fidler S., Ward H. (2014). Adherence to antiretroviral therapy in adolescents living with HIV: Systematic review and meta-analysis. Aids.

[2] Zhou S., Cluver L., Shenderovich Y., Toska E. (2021). Uncovering ART adherence inconsistencies: An assessment of sustained adherence among adolescents in South Africa. J. Int. AIDS Soc..

[3] Joint United Nations Programme on HIV/AIDS (2024). 2024 Global AIDS Report—The Urgency of Now: AIDS at a Crossroads.

[4] World Health Organization, Joint United Nations Programme on HIV/AIDS (2025). Practical Approaches and Case-Based Models for Reaching Men and Boys with Integrated HIV Services.

[5] Crocker B.C., Pit S.W., Hansen V., John-Leader F., Wright M.L. (2019). A positive approach to adolescent sexual health promotion: A qualitative evaluation of key stakeholder perceptions of the Australian Positive Adolescent Sexual Health (PASH) Conference. BMC Public Health.

[6] van Wyk B.E., Davids L.-A.C. (2019). Challenges to HIV treatment adherence amongst adolescents in a low socio-economic setting in Cape Town. South. Afr. J. HIV Med..

[7] Benyumiza D., Amongin J.F., Ochaba I., Adupa M., Abuch N., Banula C.B., Udho S. (2021). Factors Associated with Utilization of HIV Testing Services among Adolescents Aged 10–19 Years in Lira District, Northern Uganda: A Cross-Sectional Study. BioMed Res. Int..

[8] Opito R., Mpagi J., Bwayo D., Okello F., Mugisha K., Napyo A. (2020). Treatment outcome of the implementation of HIV test and treat policy at The AIDs Support Organization (TASO) Tororo clinic, Eastern Uganda: A retrospective cohort study. PLoS ONE.

[9] Chola M., Sikazwe I., Robalo M., Oduro-Bonsrah P., Coutinho A., Sheneberger R., Ozoemene J., M’pele P., Nyamweya D., Stevenson S. (2025). Africa’s defining moment: The time to lead the HIV response is now. Lancet Glob. Health.

[10] Moyo E., Moyo P., Mangwana H., Murewanhema G., Dzinamarira T. (2025). Facilitators and Barriers to Antiretroviral Therapy Adherence Among Adolescents and Young Adults in Sub-Saharan Africa: A Scoping Review. Adolescents.

[11] Chachu K.H., Maboe K.A. (2025). Strategies for strengthening same-day ART initiation, tracing people living with HIV lost to follow-up and viral load monitoring mechanisms in Ethiopia. HIV Med..

[12] Mukuku O., Govender K., Wembonyama S.O. (2025). Barriers and facilitators to HIV viral load suppression among adolescents living with HIV in Lubumbashi, Democratic Republic of the Congo: A qualitative study. PLoS ONE.

[13] Mimiaga M.J., Kuhns L.M., Biello K.B., Tian J., Skeer M.R., Psaros C., Moitra E., Chen D., Yonko E., Mayer K.H. (2025). Positive STEPS: Enhancing Medication Adherence and Achieving Viral Load Suppression in Youth Living With HIV in the United States—Results From an Efficacious Stepped Care, Randomized Controlled Trial. J. Acquir. Immune Defic. Syndr..

[14] Colominas-González E., De Antonio M., Masip M., Conde M.T.M., Cardona G., Restituto D.F., Comas D., Roch M.A., López B., Torres-Bondia F.I. (2025). Complementary and alternative medicine in HIV care: Frequency of consumption, risks and interactions with antiretroviral therapy. Eur. J. Hosp. Pharm..

